# Repair of spinal cord injury by bone marrow mesenchymal stem cell-derived exosomes: a systematic review and meta-analysis based on rat models

**DOI:** 10.3389/fnmol.2024.1448777

**Published:** 2024-08-07

**Authors:** Zhongduo Ye, Yukun Zheng, Ningning Li, Huaibin Zhang, Qiangqiang Li, Xiong Wang

**Affiliations:** ^1^The First Hospital of Lanzhou University, Lanzhou, China; ^2^The First Clinical Medical College of Lanzhou University, Lanzhou, China; ^3^Lanzhou Maternal and Child Health Hospital, Lanzhou, China

**Keywords:** spinal cord injury, bone marrow mesenchymal stem cells, exosomes, rats, systematic review

## Abstract

**Objective:**

This study aims to systematically evaluate the efficacy of bone marrow mesenchymal stem cell-derived exosomes (BMSCs-Exo) in improving spinal cord injury (SCI) to mitigate the risk of translational discrepancies from animal experiments to clinical applications.

**Methods:**

We conducted a comprehensive literature search up to March 2024 using PubMed, Embase, Web of Science, and Scopus databases. Two researchers independently screened the literature, extracted data, and assessed the quality of the studies. Data analysis was performed using STATA16 software.

**Results:**

A total of 30 studies were included. The results indicated that BMSCs-Exo significantly improved the BBB score in SCI rats (WMD = 3.47, 95% CI [3.31, 3.63]), inhibited the expression of the pro-inflammatory cytokine TNF-*α* (SMD = -3.12, 95% CI [−3.57, −2.67]), and promoted the expression of anti-inflammatory cytokines IL-10 (SMD = 2.76, 95% CI [1.88, 3.63]) and TGF-*β* (SMD = 3.89, 95% CI [3.02, 4.76]). Additionally, BMSCs-Exo significantly reduced apoptosis levels (SMD = −4.52, 95% CI [−5.14, −3.89]), promoted the expression of axonal regeneration markers NeuN cells/field (SMD = 3.54, 95% CI [2.65, 4.42]), NF200 (SMD = 4.88, 95% CI [3.70, 6.05]), and the number of Nissl bodies (SMD = 1.89, 95% CI [1.13, 2.65]), and decreased the expression of astrogliosis marker GFAP (SMD = −5.15, 95% CI [−6.47, −3.82]). The heterogeneity among studies was primarily due to variations in BMSCs-Exo transplantation doses, with efficacy increasing with higher doses.

**Conclusion:**

BMSCs-Exo significantly improved motor function in SCI rats by modulating inflammatory responses, reducing apoptosis, inhibiting astrogliosis, and promoting axonal regeneration. However, the presence of selection, performance, and detection biases in current animal experiments may undermine the quality of evidence in this study.

## 1 Background

Spinal cord injury (SCI) is one of the most severe neurological disorders, with over 27 million individuals affected worldwide and a continually increasing disease burden (GBD 2016 Neurology Collaborators, 2019). Following a primary traumatic event, secondary injury events ensue, including but not limited to ischemia, hemorrhage, blood-spinal cord barrier disruption, edema, and oxidative stress. These factors accelerate neuronal necrosis and axonal degeneration (Aslan et al., 2009; Haque et al., 2017; Anjum et al., 2020). Current treatments for SCI primarily involve surgery, medication, hyperbaric oxygen therapy, and physical therapy (Flack et al., 2022). However, due to poor plasticity of the central nervous system and the limited regenerative capacity of neurons, no effective treatments can fully restore neurological function after injury (de Almeida et al., 2023). Consequently, exploring new therapeutic approaches is of paramount importance.

In recent years, mesenchymal stem cells (MSCs) have garnered significant attention due to their multidirectional differentiation potential, low immunogenicity, of isolation and expansion, and paracrine potential (Vismara et al., 2017; He et al., 2021). MSCs exert therapeutic effects by mitigating blood-spinal cord barrier damage and neuronal apoptosis, promoting angiogenesis and axonal regeneration, and inhibiting astrogliosis and inflammatory responses (O'Shea et al., 2017; Lv et al., 2021). However, further research has revealed several limitations of MSCs, including limited targeting ability, low transplant survival rates, immune rejection, genetic variation, involvement in tumor formation, and ethical concerns (Herberts et al., 2011; Liu et al., 2019; Andrzejewska et al., 2021; Shang et al., 2022b). Additionally, the proliferation of glial cells forming scars hinders the integration, differentiation, and axonal growth of MSCs in the lesioned area (Clifford et al., 2023; Huang et al., 2023). Recent evidence increasingly suggests that the therapeutic effects of MSCs are primarily due to the release of trophic factors through paracrine mechanisms, particularly exosomes, which promote neural regeneration by modulating the inflammatory microenvironment rather than by differentiating and replacing lost cells at the injury site (Maldonado-Lasunción et al., 2018; Liau et al., 2020). Key signaling pathways such as MyD88/TRAF6 and IRF5, extracellular matrix components (e.g., type IV collagen, fibronectin, laminin), and molecules like STAT3 and PTEN also play significant roles. These molecules and pathways collectively participate in the various mechanisms by which exosomes contribute to SCI repair (Li D. et al., 2018; Chang et al., 2021; Fan et al., 2021). Exosomes offer similar therapeutic benefits to direct MSC transplantation without eliciting multiple adverse reactions, making them a potential substitute for stem cell transplantation (Zhang et al., 2018; Xian et al., 2019). Compared to MSCs, MSC-derived exosomes (MSCs-Exo) possess unique advantages, including the inability to self-replicate (thus reducing tumorigenic risk), nano-scale size facilitating blood–brain barrier penetration, high biocompatibility and low immunogenicity, widespread availability, ease of isolation and storage, and engineering capabilities. These features make MSCs-Exo more effective than MSCs in repairing SCI (Phinney and Pittenger, 2017; Zhang et al., 2019; Guy and Offen, 2020; Milbank et al., 2021; Watanabe et al., 2021; Zhuang et al., 2021).

Currently, BMSCs-Exo is a key focus in spinal cord injury (SCI) repair research and has been extensively studied in animal models of SCI. However, there is a lack of systematic reviews/meta-analyses (SRs/MAs) summarizing its therapeutic effects. Therefore, this study aims to comprehensively investigate the role of BMSCs-Exo in SCI repair through SRs/MAs, providing the latest evidence for future research and clinical translation.

## 2 Materials and methods

This study follows the PRISMA 2020 guidelines, a set of reporting standards for systematic reviews and meta-analyses, which include a 27-item checklist and a flow diagram designed to enhance the transparency and reporting quality of the research, ensuring the reliability of the results. The study protocol has been registered on the PROSPERO website (www.crd.york.ac.uk/PROSPERO/, CRD42024531749). The registration process included creating an account, filling out a form with detailed information on the study’s title and methods, uploading relevant documents, submitting for review, and obtaining a unique registration number to ensure transparency and reproducibility. Additionally, this study established detailed inclusion and exclusion criteria based on the PICOS (Population, Intervention, Comparison, Outcome, Study design) principles, as outlined below.

### 2.1 Inclusion/exclusion criteria

#### 2.1.1 Study subjects

The inclusion criteria encompass rat models of SCI, without restriction on the breed of the animal or the method of injury induction. Rats are the most commonly used animal models for SCI due to their injury and repair mechanisms being more similar to humans, standardized modeling procedures, and lower costs (Kjell and Olson, 2016).

#### 2.1.2 Interventions

The intervention includes exosomes derived from BMSCs, with no restrictions on the route of administration or dosage of the exosomes.

#### 2.1.3 Control measures

Administer the same volume of saline or phosphate-buffered saline as given to the experimental group, or simply perform the modeling without administering any medication.

#### 2.1.4 Outcome measures

1. *Basso-Beattie-Bresnahan (BBB) locomotor function score*: The BBB score is a standard scale widely used to assess the degree of motor function recovery in SCI rats, ranging from 0 (complete paralysis) to 21 (normal function) (Basso et al., 1996).
2. *Inflammatory response*: Evaluation of the expression levels of pro-inflammatory factors TNF-*α* and anti-inflammatory factors IL-10 and TGF-*β*.
3. *Apoptosis levels*: Detection of the extent of apoptosis in spinal cord tissue using TUNEL staining.
4. *Neuroregeneration and astrogliosis*: Assessment of neuroregeneration-related markers (such as NeuN, NF200, and Nissl body counts) and astrogliosis-related markers (such as GFAP).

#### 2.1.5 Study type

Inclusion of randomized controlled trials, without restriction on whether allocation concealment or blinding was implemented.

### 2.2 Data sources

Under the guidance of experienced librarians, PubMed, Embase, Web of Science, and Scopus databases were searched using a combination of subject terms and free terms. The search period ranged from the inception of the databases to March 2024. The search terms used were as follows: “bone marrow mesenchymal stem cell*” OR “BMSCs” OR (“bone” AND “mesenchymal stem cells”) AND (“exosome*” OR “exosomes” OR “exosomal” OR “extracellular vesicle” OR “extracellular vesicles” OR “extracellular particle” OR “extracellular particles” OR “microvesicle” OR “microvesicles” OR “Shedding Microvesicle” OR “Shedding Microvesicles” OR “Secretory Vesicle” OR “Secretory Vesicles” OR “Cell-Derived Microparticle” OR “Cell-Derived Microparticles” OR “microbubble” OR “microbubbles” OR “apoptotic body” OR “apoptosis bodies”) AND (“Spinal cord injury” OR “Spinal injury” OR “Spinal Cord Trauma” OR “Spinal Cord Transection” OR “Spinal Cord Laceration” OR “Post-Traumatic Myelopathy” OR “Spinal Cord Contusion”). The detailed search process for each database is provided in Supplementary Table S1.

### 2.3 Literature screening and data extraction

Two trained researchers independently screened the literature and extracted data according to the inclusion/exclusion criteria, with cross-verification. If two researchers have a disagreement regarding the inclusion/exclusion of a particular study or the extraction of certain information, a third researcher will assist in making the final judgment. Initially, researchers reviewed titles and abstracts to preliminarily select literature that met the inclusion criteria, followed by full-text retrieval for further confirmation. Information was then extracted based on a pre-designed data extraction form, including the following: 1) Basic characteristics of included studies: Author, publication year, country, study type, species of rats, gender, weight, age, sample size of experimental and control groups, model type, source of exosomes, diameter, transplantation route, and transplantation dosage. 2) Outcome measures: BBB score, inflammatory response, apoptosis levels, neuroregeneration, and astrogliosis. 3) Key elements for assessing risk of bias.

### 2.4 Risk of bias assessment

Two trained researchers independently assessed the risk of bias in the included studies using the SYRCLE risk of bias tool for animal studies. This tool is an assessment tool specifically designed for animal studies to systematically evaluate the risk of bias within the research. It covers ten domains across six aspects: selection bias, performance bias, detection bias, attrition bias, reporting bias, and other biases. These items help determine whether there are biases in the study’s design and implementation, thereby enhancing the reliability and reproducibility of animal experiments (Hooijmans et al., 2014). Each researcher conducted their assessments independently, followed by cross-verification. Disagreements were resolved through discussion or by consulting a third party. The assessment results were categorized as “Yes,” “No,” or “Unclear,” corresponding to “low,” “high,” and “uncertain” risk of bias, respectively.

### 2.5 Statistical analysis

Statistical analyses were performed using STATA 16 software. The principle of performing a Meta-analysis in STATA software involves using the “meta” or “metan” command to combine the effect sizes and standard errors from multiple independent studies. By applying either a fixed-effects model or a random-effects model, it assesses the pooled effect size. It also utilizes heterogeneity tests (such as Q statistics and I^2^) and visual tools like forest plots to provide a comprehensive conclusion on a specific issue. When a particular outcome measure is reported in different studies, STATA software integrates these results to derive the final effect size. As all outcome measures were continuous variables, meta-analyses were conducted using standardized mean difference (SMD) or weighted mean difference (WMD). The specific approach depended on the consistency of measurement methods and reporting standards across studies.

1. *BBB score*: Due to consistent measurement methods and reporting standards, WMD was used for meta-analysis.
2. *Inflammatory response*: The methods for measuring outcome indicators vary, including ELISA, WB, and qPCR; thus, SMD was employed for the meta-analysis.
3. *Apoptosis levels*: Despite all studies using TUNEL staining, the reporting methods varied (e.g., number of apoptotic cells vs. percentage), so SMD was used for meta-analysis.
4. *Neuroregeneration and astrogliosis*: Due to varied measurement methods (e.g., immunohistochemistry, immunofluorescence staining, WB, HE staining), SMD was used for meta-analysis.

Heterogeneity among study results was assessed using the *χ*^2^ test and quantified with the I^2^ statistic. If I^2^ < 50% and *p* > 0.05, indicating acceptable heterogeneity, a fixed-effects model was used for meta-analysis. Otherwise, subgroup or sensitivity analyses were conducted to explore sources of heterogeneity. If heterogeneity could not be resolved, a random-effects model was used. The significance level was set at *α* = 0.05.

## 3 Results

### 3.1 Literature search results

**Figure 1:**
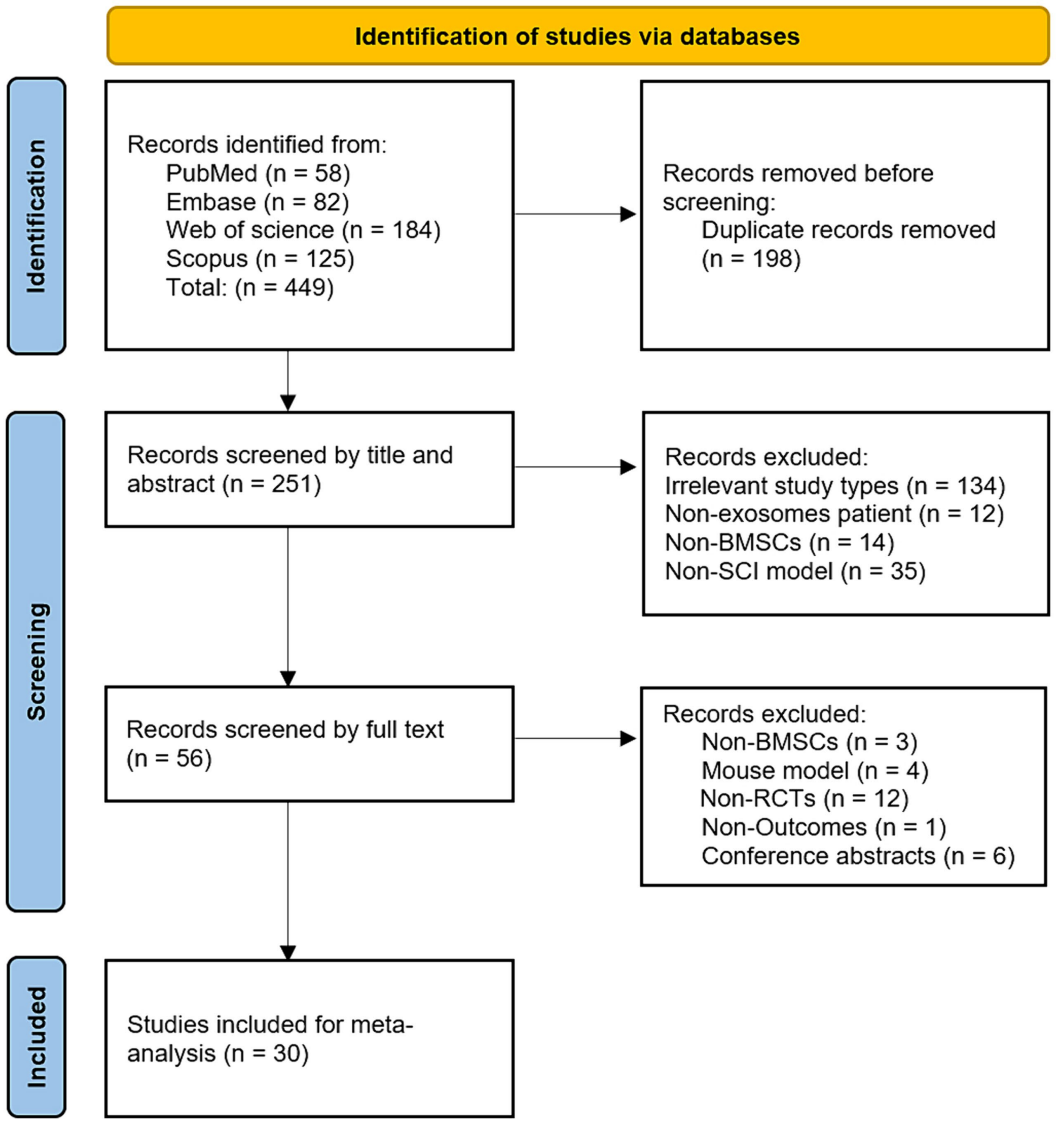
Flowchart of literature screening.

A preliminary search identified 449 animal studies on BMSCs-Exo treatment for SCI. After removing 198 duplicate records, 251 articles remained. A preliminary screening of titles and abstracts led to the exclusion of 195 articles that did not meet the inclusion criteria. The full texts of the remaining 56 articles were then reviewed in detail, resulting in the inclusion of 30 studies that met the standards (Huang et al., 2017; Wang et al., 2018; Li et al., 2019; Liu et al., 2019; Lu et al., 2019; Yu et al., 2019; Zhao et al., 2019; Zhou et al., 2019; Gu et al., 2020; Li et al., 2020; Chang et al., 2021; Chen et al., 2021; Cheng et al., 2021; Han et al., 2021; Huang et al., 2021; Luo et al., 2021; Nakazaki et al., 2021; Nie and Jiang, 2021; Zhang et al., 2021; Jia et al., 2021a,b; He et al., 2022; Wang et al., 2022; Zhou et al., 2022; Lu et al., 2023; Nakazaki et al., 2023; Shao et al., 2023; Liang et al., 2024; Tang et al., 2024; Yang et al., 2024). The literature screening process is illustrated in Figure 1.

### 3.2 Basic information of included studies

All 30 included studies were randomized controlled trials, published between 2017 and 2024. The species of rats used included Sprague–Dawley rats (25 studies) and Wistar rats (5 studies). Sixteen studies used male rats, nine used female rats, and five did not report the sex of the rats. The rats’ weights ranged from 150 g to 260 g, with seven studies not reporting the weights. The ages of the rats ranged from 6 to 12 weeks, with seven studies not reporting the ages. Sample sizes ranged from 8 to 80 rats. The SCI was located at the T10 segment in all studies. Of these, 26 studies used a spinal cord contusion model, 2 used a spinal cord hemisection model, 1 used a spinal cord compression model, and 1 used a spinal cord transection model. BMSCs were all derived from allogeneic rat bone marrow tissue. The diameter of exosomes ranged from 20 to 200 nm, with nine studies not reporting the diameter. Exosome transplantation routes included tail vein injection (26 studies), intrathecal injection (2 studies), subdural transplantation (1 study), and hydrogel-loaded implantation at the lesion site (1 study). Exosome transplantation doses ranged from 100 to 200 μg, with three studies not reporting the dosage. The basic information of the included studies is detailed in Table 1.

**Table 1:** The basic information of the included studies.

No.	Author	Year	Country	Type of study	Species	Gender	Body weight	Age	Sample size	Injury segment	Types of models	Types of exosomes	Source of exosomes	Exosome diameter	Transplantation way	Transplant dose
1	He	2022	China	RCT	SD rat	–	220 g	6 weeks	10/10	T10	Contusion	BMSCs	Bone marrow of SD rat	–	Tail veins	100 μg
2	Liang	2024	China	RCT	SD rat	Male	200–260 g	6–8 weeks	20/20	T10	Contusion	BMSCs	Bone marrow of SD rat	52.25–149.25 nm	Tail veins	100 μg
3	Chang	2021	China	RCT	SD rat	Male	220–260 g	Adult	12/12	T10	Contusion	BMSCs	Bone marrow of SD rat	30–150 nm	Intrathecal injection	–
4	Zhou	2019	China	RCT	Wistar rat	Male	200–250 g	Adult	4/4	T10	Spinal cord hemisection	BMSCs	Bone marrow of Wistar rat	40–160 nm	Tail veins	100 μg
5	Shao	2023	China	RCT	SD rat	–	–	6–8 weeks	12/12	T10	Contusion	BMSCs	Bone marrow of SD rat	–	Tail veins	200 μg
6	Yu	2019	China	RCT	SD rat	Female	230–250 g	–	20/20	T10	Contusion	BMSCs	Bone marrow of SD rat	–	Tail veins	100 μg
7	Chen	2021	China	RCT	SD rat	Female	–	6 weeks	6/6	T10	Oppression	BMSCs	Bone marrow of SD rat	50–100 nm	Tail veins	200 μg
8	Lu	2023	China	RCT	SD rat	Female	–	6 weeks	8/8	T10	Contusion	BMSCs	Bone marrow of SD rat	30–200 nm	Tail veins	100 μg
9	Luo	2020	China	RCT	SD rat	Female	170–220 g	12 weeks	10/10	T10	Contusion	BMSCs	Bone marrow of SD rat	50–150 nm	Tail veins	200 μg
10	Wang	2018	China	RCT	SD rat	Male	200–250 g	Adult	25/25	T10	Contusion	BMSCs	Bone marrow of SD rat	30–150 nm	Tail veins	200 μg
11	Li	2019	China	RCT	SD rat	Male	–	–	10/10	T10	Contusion	BMSCs	Bone marrow of SD rat	–	Tail veins	100 μg
12	Li	2019	China	RCT	Wistar rat	Male	150–200 g	Adult	50/50	T10	Contusion	BMSCs	Bone marrow of Wistar rat	–	Tail veins	200 μg
13	Cheng	2021	China	RCT	SD rat	–	–	Adult	6/6	T10	Contusion	BMSCs	Bone marrow of SD rat	30–150 nm	Hydrogel loading	200 μg
14	Yang	2023	China	RCT	SD rat	Male	200–260 g	6–8 weeks	30/30	T10	Contusion	BMSCs	Bone marrow of SD rat	30–150 nm	Tail veins	100 μg
15	Nakazaki	2023	USA	RCT	SD rat	Male	190–225 g	Adult	12/12	T10	Contusion	BMSCs	Bone marrow of SD rat	60–200 nm	Tail veins	200 μg
16	Gu	2020	China	RCT	SD rat	Male	220–260 g	Adult	10/10	T10	Contusion	BMSCs	Bone marrow of SD rat	30–150 nm	Tail veins	200 μg
17	Zhao	2019	China	RCT	Wistar rat	Male	200–250 g	Adult	23/23	T10	Spinal cord hemisection	BMSCs	Bone marrow of Wistar rat	20–150 nm	Tail veins	100 μg
18	Jia	2019	China	RCT	SD rat	Male	200–250 g	Adult	25/25	T10	Contusion	BMSCs	Bone marrow of SD rat	–	Tail veins	200 μg
19	Wang	2022	China	RCT	Wistar rat	Female	–	–	10/10	T10	Contusion	BMSCs	Bone marrow of Wistar rat	–	Intrathecal injection	–
20	Nakazaki	2021	USA	RCT	SD rat	Male	185–215 g	Adult	17/14	T10	Contusion	BMSCs	Bone marrow of SD rat	25–140 nm	Tail veins	–
21	Nie	2021	China	RCT	SD rat	–	190–220 g	–	24/24	T10	Transection	BMSCs	Bone marrow of SD rat	50–150 nm	Tail veins	100 μg
22	Huang	2017	China	RCT	SD rat	Male	180–220 g	Adult	15/15	T10	Contusion	BMSCs	Bone marrow of SD rat	20–130 nm	Tail veins	100 μg
23	Liu	2018	China	RCT	SD rat	Female	170–220 g	Adult	10/10	T10	Contusion	BMSCs	Bone marrow of SD rat	20–150 nm	Tail veins	200 μg
24	Zhou	2022	China	RCT	SD rat	Female	200–250 g	12 weeks	40/40	T10	Contusion	BMSCs	Bone marrow of SD rat	Average 100 nm	Tail veins	200 μg
25	Jia	2021	China	RCT	SD rat	Male	230–250 g	–	10/10	T10	Contusion	BMSCs	Bone marrow of SD rat	–	Tail veins	120 μg
26	Zhang	2021	China	RCT	SD rat	Male	200–230 g	8 weeks	8/8	T10	Contusion	BMSCs	Bone marrow of SD rat	Average 99.02 nm	Tail veins	200 μg
27	Han	2021	China	RCT	Wistar rat	Female	200–250 g	6–8 weeks	8/8	T10	Contusion	BMSCs	Bone marrow of Wistar rat	Average 100 nm	Subdural	100 μg
28	Tang	2024	China	RCT	SD rat	–	–	–	6/6	T10	Contusion	BMSCs	Bone marrow of SD rat	70–120 nm	Tail veins	120 μg
29	Huang	2021	China	RCT	SD rat	Female	200–250 g	10 weeks	20/20	T10	Contusion	BMSCs	Bone marrow of SD rat	30–200 nm	Tail veins	100 μg
30	Jia	2019	China	RCT	SD rat	Male	230–250 g	–	10/10	T10	Contusion	BMSCs	Bone marrow of SD rat	–	Tail veins	120 μg

### 3.3 Risk of bias assessment results

**Figure 2:**
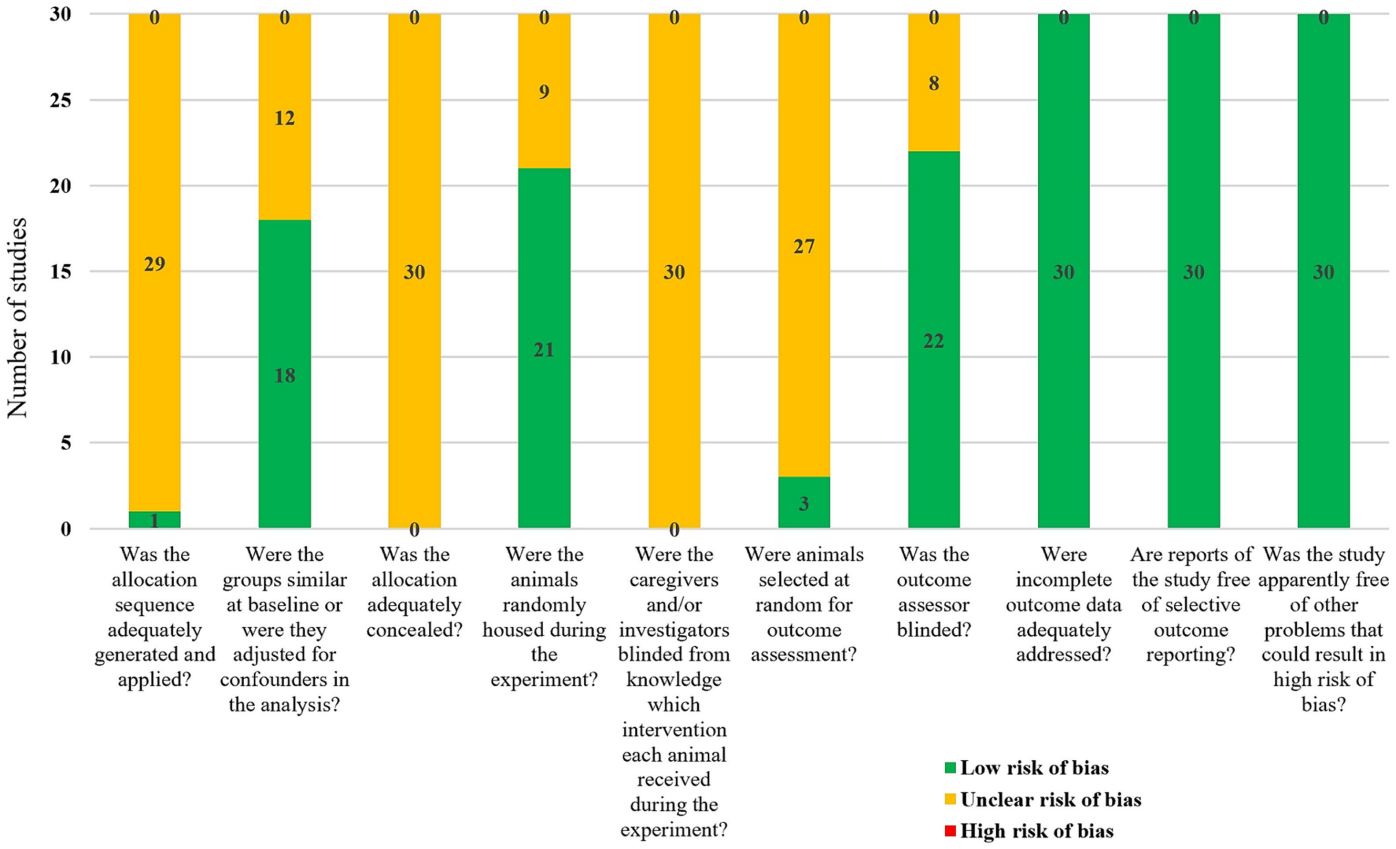
Meta-analysis results of bias risk assessment in included studies.

Although all 30 included studies were randomized controlled trials, only one study explicitly reported the specific method of randomization (random number table), while the remaining 29 studies did not clarify their randomization methods. Eighteen studies reported similar baseline characteristics, such as sex, weight, and age of the rats. None of the studies explicitly reported whether blinding was implemented for animal caretakers and/or researchers. However, 21 studies reported randomizing the placement of animals during the experiment. Only three studies randomly selected animals for outcome assessment, while 22 studies employed blinding during the measurement or evaluation of outcomes. No animals died or were lost during the modeling and treatment periods. Although study protocols were not available, all studies clearly reported their expected outcomes. The results of the risk of bias assessment are detailed in Figure 2.

### 3.4 Meta-analysis results

#### 3.4.1 BBB scores

**Figure 3:**
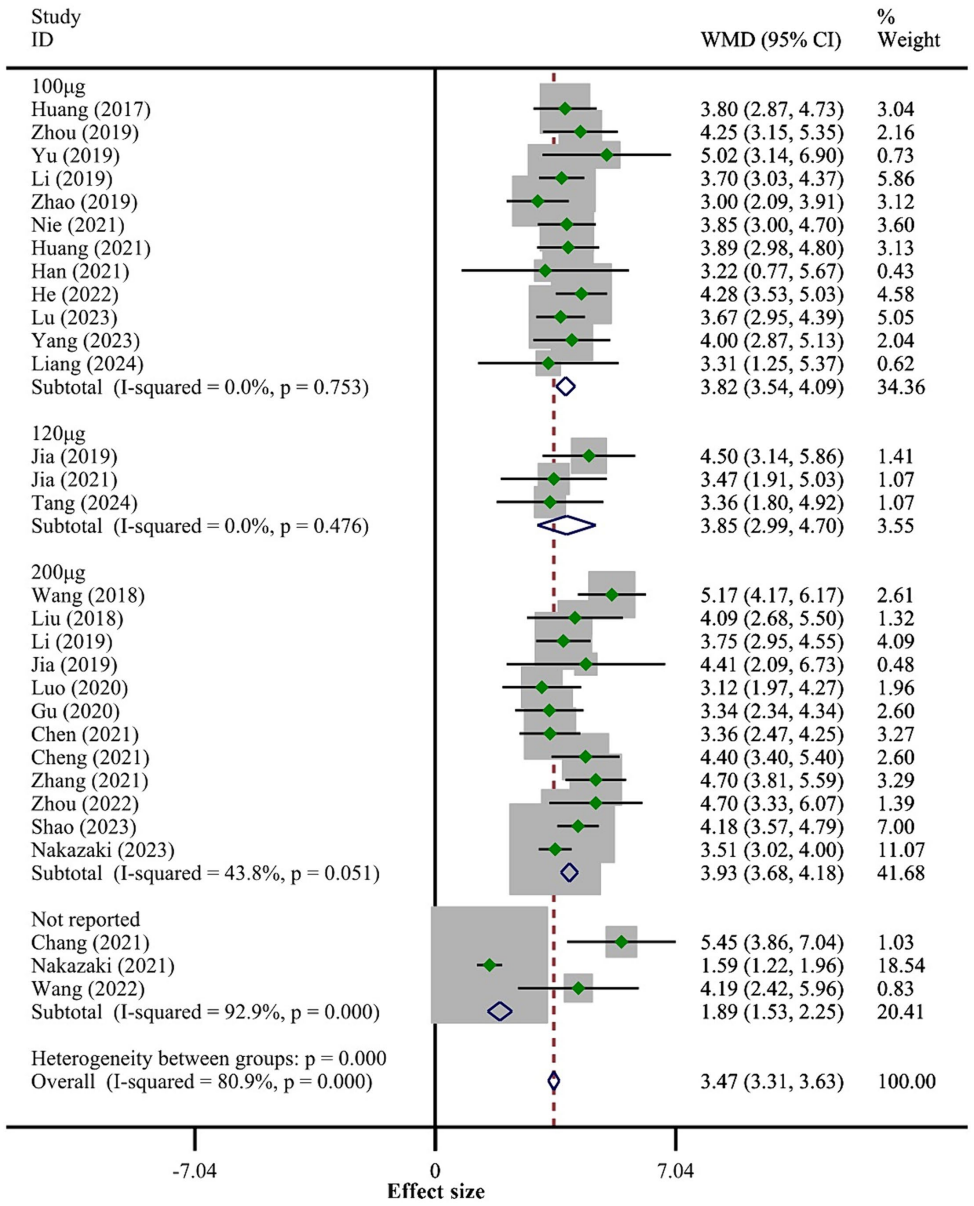
Meta-analysis results of BBB score.

All 30 studies reported BBB scores. The fixed-effect model meta-analysis indicated that rats in the BMSCs-Exo group had significantly higher BBB scores compared to the placebo group (WMD = 3.47, 95% CI [3.31, 3.63]), suggesting that BMSCs-Exo significantly improve motor function in rats with SCI. However, the I-squared value was 80.9% and *p* = 0, indicating substantial heterogeneity among the studies. Therefore, we performed a subgroup analysis based on exosome transplantation dose. We categorized the doses into four groups: 100 μg, 120 μg, 200 μg, and an unreported dose group. The subgroup analysis showed a significant reduction in heterogeneity, with I-squared values in all subgroups falling below 50%, suggesting that exosome transplantation dose was the primary source of heterogeneity. Additionally, the subgroup analysis revealed a positive correlation between the exosome transplantation dose and BBB scores, as shown in Figure 3.

#### 3.4.2 Inflammatory response

**Figure 4:**
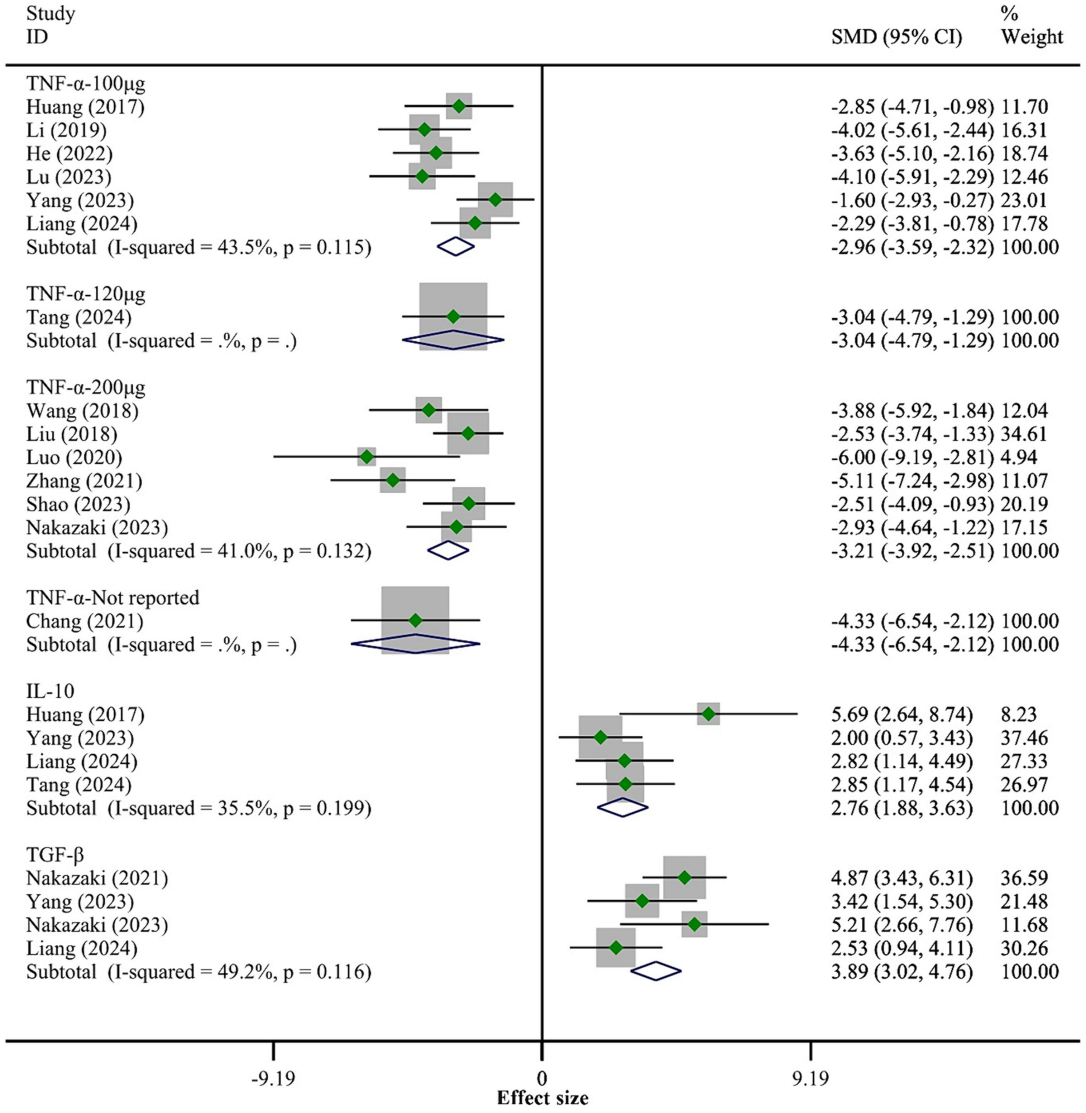
Meta-analysis results of the inflammatory response.

A total of 14 studies reported the expression levels of TNF-*α*. The fixed-effect model meta-analysis showed that the BMSCs-Exo group had significantly lower TNF-*α* expression levels compared to the placebo group (SMD = −3.12, 95% CI [−3.57, −2.67]), indicating that BMSCs-Exo significantly inhibited the expression of pro-inflammatory factors (the overall meta-analysis results are not shown in Figure 4). Due to the high heterogeneity among the studies (I-squared = 75.3%, *p* = 0.032), we conducted a subgroup analysis based on the exosome transplantation dose. The subgroup analysis showed that heterogeneity was significantly reduced, with I-squared values in all subgroups being below 50%, indicating that the exosome transplantation dose was the primary source of heterogeneity. Furthermore, the subgroup analysis showed that as the exosome transplantation dose increased, the expression levels of TNF-*α* in rats gradually decreased, as shown in Figure 4.

Five studies reported the expression levels of IL-10 and TGF-*β*. The meta-analysis results indicated that compared to the placebo group, the BMSCs-Exo group had significantly higher expression levels of IL-10 (SMD = 2.76, 95% CI [1.88, 3.63]) and TGF-*β* (SMD = 3.89, 95% CI [3.02, 4.76]), suggesting that BMSCs-Exo significantly promoted the expression of anti-inflammatory factors post-SCI. See Figure 4 for details.

#### 3.4.3 Apoptosis levels

**Figure 5:**
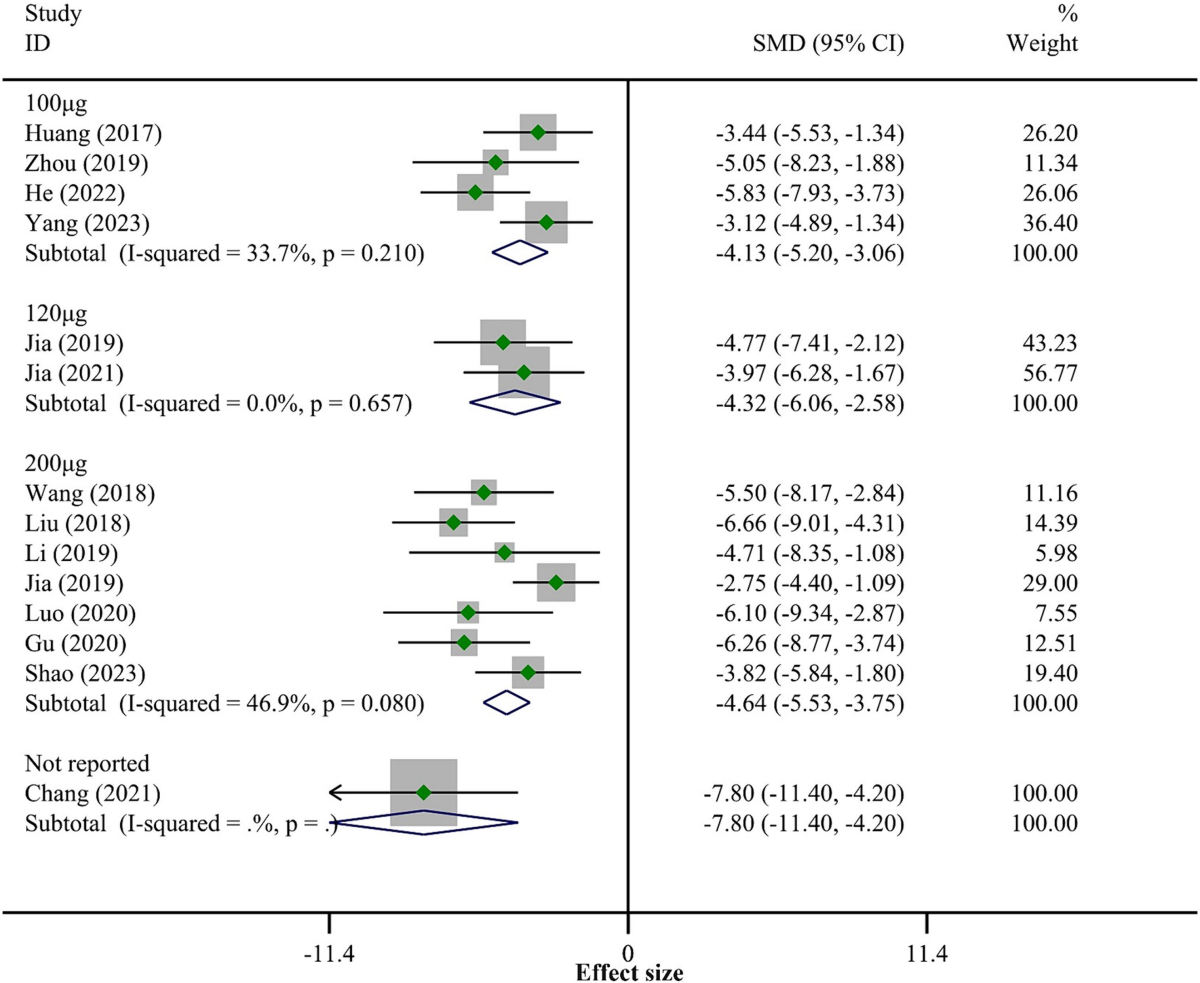
Meta-analysis results of apoptosis levels.

A total of 14 studies reported the levels of apoptosis in SCI tissue. The fixed-effect model meta-analysis showed that the BMSCs-Exo group had significantly lower levels of apoptosis compared to the placebo group (SMD = −4.52, 95% CI [−5.14, −3.89]), indicating that BMSCs-Exo significantly inhibited the extent of apoptosis post-SCI (the overall meta-analysis results are not shown in Figure 5). Although there was considerable heterogeneity among the studies (I-squared = 47.3%, *p* = 0.102), it was within an acceptable range (I-squared <50%). Nevertheless, we performed a subgroup analysis based on the exosome transplantation dose to explore its impact on apoptosis. The subgroup analysis showed that as the exosome transplantation dose increased, the level of apoptosis in rat spinal cord tissue significantly decreased. See Figure 5 for details.

#### 3.4.4 Neural regeneration and astrogliosis

**Figure 6:**
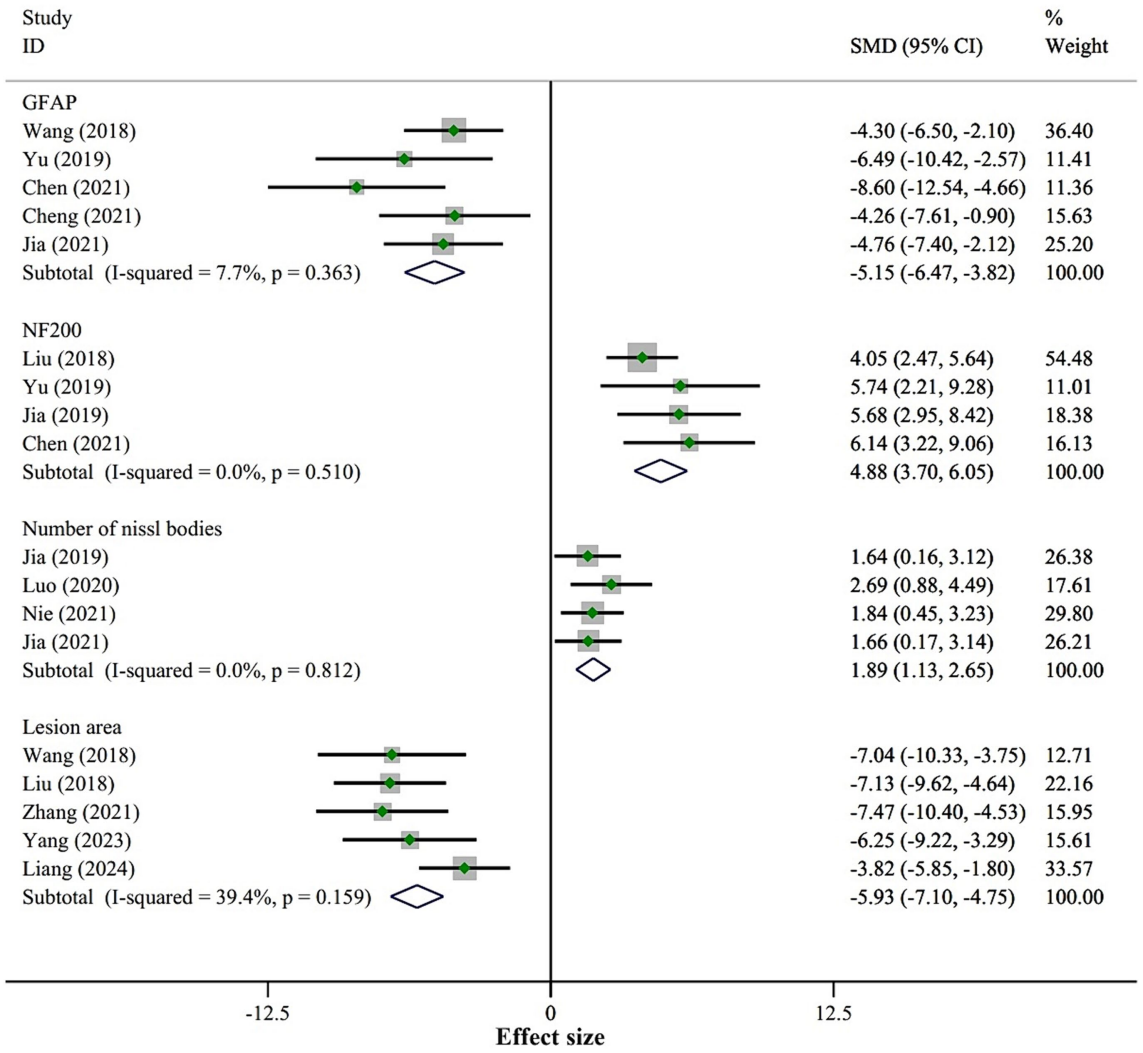
Meta-analysis results of nerve regeneration and astrogliosis.

We included three outcome measures related to neural regeneration: NeuN cells/field (5 studies), NF200 (4 studies), and the number of Nissl bodies (4 studies). The fixed-effect model meta-analysis revealed that, compared to the placebo group, the BMSCs-Exo group showed significantly higher levels of NeuN cells/field (SMD = 3.54, 95% CI [2.65, 4.42]), NF200 (SMD = 4.88, 95% CI [3.70, 6.05]), and the number of Nissl bodies (SMD = 1.89, 95% CI [1.13, 2.65]), indicating that BMSCs-Exo significantly promoted neural regeneration after SCI. Detailed results are presented in Figure 6.

For astrogliosis, we included one outcome measure, GFAP. The fixed-effect model meta-analysis demonstrated that the expression level of GFAP in the BMSCs-Exo group was significantly lower compared to the placebo group (SMD = −5.15, 95% CI [−6.47, −3.82]), suggesting that BMSCs-Exo significantly inhibited astrogliosis after SCI. See Figure 6 for detailed results.

Additionally, the meta-analysis of the lesion area showed that BMSCs-Exo significantly reduced the lesion area in SCI rats (SMD = −5.93, 95% CI [−7.10, −4.75]). Detailed results are presented in Figure 6.

#### 3.4.5 Publication bias detection

Generally, detecting publication bias is meaningful when there are 10 or more studies reporting an outcome measure. Therefore, we performed a publication bias test for the BBB score outcome measure. The results indicated that the funnel plot was asymmetric, suggesting the potential presence of publication bias in the current research field. Please refer to Supplementary Figure S1.

## 4 Discussion

Following SCI, a series of secondary pathophysiological changes occur, including inflammation and neuronal apoptosis at the injury site, which subsequently leads to the formation of cavities and astrocytic scars, inhibiting axonal regeneration. Therefore, reversing these pathological processes is crucial for promoting SCI repair. For SCI patients, the restoration of sensory and motor functions is a core goal to improve their quality of life (Shang et al., 2022b). Our meta-analysis, based on 30 randomized controlled trials, indicates that BMSCs-Exo significantly enhances motor function in rats post-SCI. This strongly demonstrates the therapeutic potential of BMSCs-Exo. Previous meta-analysis has shown that exosomes can significantly improve motor function in SCI animals (Yi and Wang, 2021; Shang et al., 2024). Our study, however, focuses more specifically on exosomes derived from BMSCs-Exo and includes only rat SCI models, thereby reducing inter-study heterogeneity and making the results more reliable. Additionally, we explored the effects of BMSCs-Exo on inflammation, apoptosis, neural regeneration, and astrogliosis—important areas that previous studies focusing solely on motor function had not addressed.

Apoptosis and inflammatory responses are major events in secondary SCI. Neuronal apoptosis plays a critical role in the functional outcomes and prognosis of SCI, and it is primarily regulated by the upstream Bcl-2 family and downstream caspase family. Among these, the anti-apoptotic protein Bcl-2 and the pro-apoptotic protein Bax are common markers of apoptosis (Huang et al., 2017). Numerous studies have shown that BMSCs-Exo can significantly reduce the expression levels of apoptotic proteins such as Bax, cleaved caspase-3, and cleaved caspase-9, while promoting the expression of the anti-apoptotic protein Bcl-2 (Adams and Cory, 1998; Lin et al., 2006; Li et al., 2019). These findings are consistent with the results of our meta-analysis, which revealed a significant reduction in neuronal apoptosis rates in injured spinal cord tissue following BMSCs-Exo treatment. These results, in combination with BBB scores, suggest that BMSCs-Exo facilitates the recovery of motor function by inhibiting neuronal apoptosis (Li et al., 2019). The activation of the Wnt/*β*-Catenin signaling pathway plays a crucial role in suppressing neuronal apoptosis after acute SCI (Gao et al., 2015; Li X. et al., 2018). Li et al. (2019) discovered that BMSCs-Exo inhibits cell apoptosis by activating the Wnt/*β*-Catenin signaling pathway, thereby reducing the protein expression levels of caspase-3 and caspase-9 in neurons, and increasing the expression of Bcl-2. Additionally, BMSCs-Exo activates cellular autophagy by upregulating autophagy-related proteins (such as LC3IIB), reducing neuronal apoptosis, and promoting neural function recovery (Gu et al., 2020). However, the specific mechanisms remain unclear. Some studies have found that BMSCs-Exo enhances autophagy-related protein expression and inhibits NLRP3 inflammasome activation in macrophages/microglia through the miR-21a-5p/PELI1 axis. This can improve motor function recovery and alleviate neuroinflammation following SCI (Gu et al., 2024).

The inflammatory response is also crucial in the onset and progression of SCI (Xu et al., 2018). Inflammation is a significant cause of secondary SCI, further impairing neuronal function and exacerbating neural cell damage. The inflammatory response is intensified by the release of immune cells and inflammatory factors (Anjum et al., 2020). After SCI, the blood–brain barrier is compromised, leading to the infiltration of neutrophils into the spinal cord area. Neutrophils, as the earliest circulating immune cells to arrive at the injury site during the acute phase, release proteases such as elastase and myeloperoxidase through degranulation, along with reactive oxygen species. These substances damage spinal cord tissue and contribute to the formation of fibrotic scars, which hinder axonal regeneration (Dolma and Kumar, 2021). Moreover, neutrophils can upregulate the expression of inflammatory cytokines like TNF-*α* and IL-1*β*, exacerbating neuronal damage (Pan et al., 2002; Han et al., 2015). Based on a comprehensive analysis of previous research results, BMSCs-Exo significantly reduces the expression levels of the pro-inflammatory cytokine TNF-*α* in spinal cord injury tissues while markedly increasing the expression levels of the anti-inflammatory cytokines IL-10 and TGF-*β* (He et al., 2022; Liang et al., 2024; Yang et al., 2024).This demonstrates the potent regulatory capability of BMSCs-Exo on the post-SCI inflammatory microenvironment. The regulation of the inflammatory response in SCI involves the modulation of inflammatory cells such as microglia, astrocytes, and macrophages, as well as pro-inflammatory cytokines. Tail vein injection of BMSCs-Exo can significantly downregulate levels of pro-inflammatory cytokines like TNF-*α* and IL-1*β*, while upregulating levels of anti-inflammatory cytokines like IL-10, thereby exerting an anti-inflammatory effect (Huang et al., 2017). However, the efficacy of BMSCs-Exo may vary depending on the dosage and administration route. Currently, there is a lack of clear evidence regarding the differences in efficacy between high and low doses of BMSCs-Exo. Additionally, different administration routes, such as intrathecal or intravenous injection, may affect the distribution and targeting of BMSCs-Exo in the injured spinal cord. Therefore, future research should focus on optimizing dosage and administration routes to maximize the therapeutic effects of BMSCs-Exo. Additionally, BMSCs-Exo exerts anti-inflammatory and neuroprotective effects by downregulating the phosphorylation of the NF-κB P65 subunit, thereby inhibiting the differentiation of astrocytes into the A1 phenotype (Wang et al., 2018; Liu et al., 2019; Wu et al., 2021). BMSCs-Exo also mitigates the inflammatory response in SCI rats by inhibiting the expression of TLR4 downstream genes MyD88 and TRAF6, thereby suppressing the release of NF-κB (Fan et al., 2021). Furthermore, BMSCs-Exo enhances neuronal regeneration and improves motor function in SCI rats by inhibiting the expression of caspase 1 and IL-1*β*, thereby reducing pyroptosis (Zhou et al., 2022). Moreover, BMSCs-Exo provides protective effects against SCI by binding to M2-type microglia, inhibiting the synthesis and release of complement mRNA and the activation of NF-κB (Lankford et al., 2018; Zhao et al., 2019). Chang et al. (2021) found that intervention with BMSCs-Exo in SCI model mice targets and negatively regulates IRF5 to inhibit the differentiation of microglia into the M1 phenotype and the secretion of inflammatory factors. Other studies have pointed out that intravenously injected BMSCs-Exo can be taken up by M2-type macrophages at the injury site, thereby modulating the inflammatory response at the injury site. The mechanism involves increasing the secretion of anti-inflammatory factors and preventing the transformation of M2-type macrophages into the M1 type (Lo Sicco et al., 2017; Lankford et al., 2018; Wang et al., 2018). These studies indicate that BMSCs-Exo can reduce secondary injury and promote SCI repair through anti-inflammatory and anti-apoptotic mechanisms. However, its therapeutic effects are still in the experimental research stage and have not yet been explored in clinical settings.

The ultimate goal of various therapies for treating SCI is to promote the recovery of neural function. After SCI occurs, axonal rupture often happens, leading to the interruption of multiple intercellular signaling pathways, which directly affects the reconstruction of the spinal cord nervous system function. In this process, apoptotic or necrotic nerve cells release inflammatory factors, resulting in a large number of neutrophils and astrocytes congregating around the injured central nervous system (CNS) to repair the damage (Fawcett et al., 2012). These immune cells and glial cells accumulate in the CNS and secrete an excess of extracellular matrix (such as type IV collagen, fibronectin, and laminin), forming a glial scar that is difficult to remove (O'Shea et al., 2017; Lv et al., 2021). While astrogliosis is crucial for sealing off the injury site and restoring tissue integrity, it often becomes excessively prominent after SCI. The formation of glial scars can stimulate the production of GFAP, which in turn activates the RhoA signaling pathway. The RhoA signaling pathway plays a crucial role in cytoskeletal remodeling, growth cone collapse, and inhibition of axon regeneration. The growth cone is a dynamic structure at the tip of a neuron’s axon, responsible for sensing the external environment and guiding axon growth (Franze, 2020). When RhoA is activated, it affects actin fiber contraction through ROCK, leading to growth cone collapse, thereby hindering axon extension and inhibiting neuron regeneration (Karimi-Abdolrezaee and Billakanti, 2012; Tsujioka and Yamashita, 2021). Therefore, inhibiting astrogliosis as much as possible while promoting axonal regeneration is an innovative and effective strategy for repairing SCI. Li et al. found that BMSCs-Exo can activate the ERK1/2, STAT3, and CREB signaling pathways, all of which are classic pathways for neuronal and axonal regeneration (Ferrer-Molina et al., 2018). Xu et al. (2019) discovered that miRNA-19 and miRNA-21 in exosomes can regulate apoptosis and differentiation of neuronal cells in rats with spinal cord injury by targeting and inhibiting PTEN expression. However, the study did not explore the underlying mechanisms further. *In vivo* experiments demonstrated that BMSCs-Exo promote neural regeneration after SCI by activating the PI3K/AKT pathway, as evidenced by an increase in the number of Nissl bodies and high expression of NF200 in SCI tissues following BMSCs-Exo intervention (Luo et al., 2021). This is consistent with our findings, where the BMSCs-Exo group showed a significant increase in NF200 and the number of Nissl bodies compared to the groups given saline or phosphate-buffered saline. Conversely, the expression level of GFAP, a marker associated with astrogliosis, was significantly reduced. This demonstrates the significant promoting effect of BMSCs-Exo on neural regeneration and their inhibitory effect on astrogliosis. Additionally, Han et al. (2021) found that TGF-*β* in BMSCs-Exo enhances the expression of Smad6, inhibiting the excessive differentiation of neural stem cells into astrocytes and promoting neuronal regeneration. In fact, although exosomes have a significant therapeutic effect on spinal cord injury (SCI), the specific therapeutic mechanisms and targets are not yet fully understood. Currently, most studies focus on the role of miRNAs. Recently, some miRNAs such as miRNA-486, miRNA-21, and miRNA-126 have been identified as potential new targets for SCI treatment (Jee et al., 2012; Hu et al., 2013, 2015). Exosomes can penetrate the blood–brain barrier or the blood-spinal cord barrier, thereby enhancing the therapeutic effects of miRNAs (Ding et al., 2019). For example, Liu et al. found that exosomes carrying miRNA-216a-5p significantly improve therapeutic potential by inhibiting the TLR4/NF-κB pathway and activating the PI3K/Akt pathway, thus shifting microglia from a pro-inflammatory M1 phenotype to an anti-inflammatory M2 phenotype (Liu et al., 2020). Similarly, Zhou et al. (2019) demonstrated that miRNA-21-modified BMSCs-Exo significantly promote functional recovery, reduce lesion volume, and decrease apoptosis, primarily by downregulating the expression of the pro-apoptotic gene FasL. Further research [31570818] also revealed that miRNA-21 enhances cell viability and inhibits apoptosis by targeting the PTEN/PDCD4 signaling pathway. These findings not only uncover the potential mechanisms of exosomes in SCI treatment but also provide an important basis for developing new therapeutic strategies in the future.

### 4.1 Strengths and limitations

As the first research investigating the repair of spinal cord injury (SCI) using BMSCs-Exo, this study focuses on a rat SCI model, comprehensively exploring the effects of BMSCs-Exo on motor function, inflammatory response, apoptosis, glial scar formation, and axonal regeneration after SCI. This lays a foundation for further research and clinical translation of BMSCs-Exo. However, the current animal experiments have limitations in random grouping, allocation concealment, blinding, outcome measurement, and reporting, which increase the risk of selection bias, implementation bias, and measurement bias. Additionally, the inclusion of only English literature may result in language bias, and the omission of grey literature and conference abstracts may lead to publication bias. It is worth noting that due to the limited data detected and reported in the included studies, we only incorporated certain indicators related to inflammation, apoptosis, nerve regeneration, and astrocyte proliferation. In reality, there are many more indicators that could reflect the role of BMSCs in SCI, but current studies have not fully reported these, which is an area for improvement in future animal experiments.

### 4.2 Research prospects

Our subgroup analysis of different transplant doses successfully reduced heterogeneity between studies, indicating that transplant dose is a major factor affecting treatment outcomes. This aligns with findings from Shang et al. (2022a,c), who observed that higher doses of exosomes produce better therapeutic effects. Higher doses of exosomes significantly enhance SCI treatment by providing more repair factors, boosting anti-inflammatory effects, promoting angiogenesis, optimizing cell communication, and offering neurotrophic support. Additionally, the timing, frequency, and measurement of outcomes related to exosome transplantation may also impact treatment efficacy. Studies vary widely in their dosing frequency, with some using single doses and others employing multiple administrations. Multiple doses can maintain exosome concentrations at the injury site, thereby enhancing efficacy, but frequent dosing may increase treatment complexity and cost. Future research should explore the effects of different dosing frequencies on BMSCs-Exo efficacy to identify the optimal regimen. The route of administration also plays a significant role in BMSCs-Exo effectiveness. Intrathecal injection can directly target the spinal cord injury site, improving bioavailability and efficacy, whereas intravenous injection, although more convenient, may reduce exosome concentrations at the injury site due to systemic distribution (Yang et al., 2022). Future studies should compare the efficacy of different administration routes to optimize strategies. Due to limited data, further subgroup analyses are not currently feasible. There are also variations in how different studies explore the neuroprotective and reparative mechanisms of BMSCs-Exo. Some focus on modulating inflammation, reducing cell apoptosis, inhibiting glial scar formation, and promoting axon regeneration (Ren et al., 2020; Fan et al., 2021; Liu et al., 2021); while others emphasize angiogenesis and neural/glial differentiation (Gabr et al., 2015). These differences may stem from variations in study design, animal models, and exosome sources. Thus, future research needs to standardize doses, frequencies, and routes, and further investigate the specific mechanisms of BMSCs-Exo to reduce heterogeneity and improve comparability. The source of exosomes also affects therapeutic outcomes. Autologous BMSCs-Exo transplantation, derived from the patient’ s own cells, has good immunocompatibility, reducing immune rejection and enhancing treatment safety and efficacy. In contrast, allogeneic BMSCs-Exo, which are more widely available and easier to produce on a large scale, may carry a risk of immune rejection. Despite the promising effects of exosomes, further validation is needed to determine if these findings can be effectively translated to clinical practice. Overall, research on BMSCs-Exo for SCI treatment is still in the exploratory phase, with a limited number of studies primarily using rodent models, especially Sprague–Dawley rats. Given the anatomical differences between human and rodent spinal cords (e.g., differences in SCI area and neural complexity), there is a need to expand research to include larger animal models.

## 5 Conclusion

BMSCs-Exo can regulate multiple signaling pathways and reduce cytokine levels, significantly improving the inflammatory response in SCI rats, inhibiting neuron apoptosis and astrocyte proliferation, and promoting axonal regeneration, thereby enhancing motor function recovery. Higher doses of exosomes yield better therapeutic effects. However, the presence of selection bias, implementation bias, and measurement bias in animal experiments reduces the evidence quality of this study. Future research should standardize the implementation and reporting of animal experiments and conduct more high-quality studies to further explore the efficacy and mechanisms of BMSCs-Exo.

## Data availability statement

The original contributions presented in the study are included in the article/Supplementary material, further inquiries can be directed to the corresponding author.

## Author contributions

ZY: Methodology, Project administration, Software, Visualization, Writing – original draft. YZ: Formal analysis, Methodology, Resources, Software, Writing – original draft. NL: Conceptualization, Methodology, Validation, Visualization, Writing – original draft. HZ: Conceptualization, Data curation, Formal analysis, Resources, Writing – original draft. QL: Investigation, Methodology, Validation, Visualization, Writing – original draft. XW: Conceptualization, Funding acquisition, Methodology, Project administration, Writing – review & editing.
