# Supplementary material for: Repair of spinal cord injury by bone marrow mesenchymal stem cell-derived exosomes: a systematic review and meta-analysis based on rat models
Source: Front Mol Neurosci. 2024 Aug 7;17:1448777. doi: 10.3389/fnmol.2024.1448777 (PMC11335736; doi:10.3389/fnmol.2024.1448777)
Supplement: Supplementary file 1 [file Data_Sheet_1.docx]

**Repair of Spinal Cord Injury by Bone Marrow Mesenchymal Stem Cell-Derived Exosomes: A Systematic Review and Meta-Analysis Based on Rat Models**

**Supplementary Table 1: Search Strategies**

| **PubMed**  #1: "exosome*"[Title/Abstract] OR "exosomes"[Title/Abstract] OR "exosomal"[Title/Abstract] OR "extracellular vesicle"[Title/Abstract] OR "extracellular vesicles"[Title/Abstract] OR "extracellular particle"[Title/Abstract] OR "extracellular particles"[Title/Abstract] OR "microvesicle"[Title/Abstract] OR "microvesicles"[Title/Abstract] OR "Shedding Microvesicle"[Title/Abstract] OR "Shedding Microvesicles"[Title/Abstract] OR "Secretory Vesicle"[Title/Abstract] OR "Secretory Vesicles"[Title/Abstract] OR "Cell-Derived Microparticle"[Title/Abstract] OR "Cell-Derived Microparticles"[Title/Abstract] OR "microbubble"[Title/Abstract] OR "microbubbles"[Title/Abstract] OR "apoptotic body"[Title/Abstract] OR "apoptosis bodies"[Title/Abstract]  #2: "Exosomes"[MeSH Major Topic] OR "Extracellular Vesicles"[MeSH Major Topic] OR "Cell-Derived Microparticles"[MeSH Terms] OR "Microbubbles"[MeSH Major Topic]  #3: #1 OR #2  #4: "bone marrow mesenchymal stem cell*"[Title/Abstract] OR "BMSCs"[Title/Abstract] OR ("bone"[Title/Abstract] AND "mesenchymal stem cells"[Title/Abstract])  #5: "Mesenchymal Stem Cells"[MeSH Major Topic] AND "Bone Marrow"[MeSH Terms]  #6: #4 OR #5  #7: "spinal cord injury"[Title/Abstract] OR "spinal injury"[Title/Abstract] OR "spinal cord trauma"[Title/Abstract] OR "spinal cord transection"[Title/Abstract] OR "spinal cord laceration"[Title/Abstract] OR "post traumatic myelopathy"[Title/Abstract] OR "spinal cord contusion"[Title/Abstract]  #8: "Spinal Cord Injuries"[MeSH Terms]  #9: #7 OR #8  #10: #3 AND #6 AND #9  **Web of science**  ((TS=(exosome* OR exosomes OR exosomal OR “extracellular vesicle” OR “extracellular vesicles” OR “extracellular particle” OR “extracellular particles” OR “microvesicle” OR “microvesicles” OR “Shedding Microvesicle” OR “Shedding Microvesicles” OR “Secretory Vesicle” OR “Secretory Vesicles” OR “Cell-Derived Microparticle” OR “Cell-Derived Microparticles” OR “microbubble” OR “microbubbles” OR “apoptotic body” OR “apoptosis bodies”)) AND TS=(Spinal cord injury OR Spinal injury OR Spinal Cord Trauma OR Spinal Cord Transection OR Spinal Cord Laceration OR Post-Traumatic Myelopathy OR Spinal Cord Contusion)) AND TS=(bone marrow mesenchymal stem cell* OR BMSCs OR (bone AND mesenchymal stem cells))  **Embase**  #1: exosome*:ti,ab,kw OR exosomes:ti,ab,kw OR exosomal:ti,ab,kw OR 'extracellular vesicle':ti,ab,kw OR 'extracellular vesicles':ti,ab,kw OR 'extracellular particle':ti,ab,kw OR 'extracellular particles':ti,ab,kw OR 'microvesicle':ti,ab,kw OR 'microvesicles':ti,ab,kw OR 'shedding microvesicle':ti,ab,kw OR 'shedding microvesicles':ti,ab,kw OR 'secretory vesicle':ti,ab,kw OR 'secretory vesicles':ti,ab,kw OR 'cell-derived microparticle':ti,ab,kw OR 'cell-derived microparticles':ti,ab,kw OR 'microbubble':ti,ab,kw OR 'microbubbles':ti,ab,kw OR 'apoptotic body':ti,ab,kw OR 'apoptosis bodies':ti,ab,kw  #2: 'exosome'/exp  #3: 'exosomes'/exp  #4: 'extracellular vesicle'/exp  #5: 'microvesicle'/exp  #6: 'secretory vesicle'/exp  #7: 'cell-derived microparticle'/exp  #8: 'microbubble'/exp  #9: 'apoptotic body'/exp  #10: #1 OR #2 OR #3 OR #4 OR #5 OR #6 OR #7 OR #8 OR #9  #11: 'spinal cord injury':ti,ab,kw OR 'spinal injury':ti,ab,kw OR 'spinal cord trauma':ti,ab,kw OR 'spinal cord transection':ti,ab,kw OR 'spinal cord laceration':ti,ab,kw OR 'post-traumatic myelopathy':ti,ab,kw OR 'spinal cord contusion':ti,ab,kw  #12: 'spinal cord injury'/exp  #13: 'spinal injury'/exp  #14: 'spinal cord trauma'/exp  #15: 'spinal cord transection'/exp  #16: #11 OR #12 OR #13 OR #14 OR #15  #17: 'bone marrow mesenchymal stem cell*':ti,ab,kw OR bmscs:ti,ab,kw OR (bone:ti,ab,kw AND 'mesenchymal stem cells':ti,ab,kw)  #18: 'bone'/exp  #19: 'mesenchymal stem cells'/exp  #20: #18 AND #19  #21: #17 OR #20  #22: #10 AND #16 AND #21  **Scopus**  TITLE-ABS-KEY("exosome*" OR "exosomes" OR "exosomal" OR "extracellular vesicle" OR "extracellular vesicles" OR "extracellular particle" OR "extracellular particles" OR "microvesicle" OR "microvesicles" OR "Shedding Microvesicle" OR "Shedding Microvesicles" OR "Secretory Vesicle" OR "Secretory Vesicles" OR "Cell-Derived Microparticle" OR "Cell-Derived Microparticles" OR "microbubble" OR "microbubbles" OR "apoptotic body" OR "apoptosis bodies") AND TITLE-ABS-KEY("Spinal cord injury" OR "Spinal injury" OR "Spinal Cord Trauma" OR "Spinal Cord Transection" OR "Spinal Cord Laceration" OR "Post-Traumatic Myelopathy" OR "Spinal Cord Contusion") AND TITLE-ABS-KEY("bone marrow mesenchymal stem cell*" OR "BMSCs" OR ("bone" AND "mesenchymal stem cells")) |
| --- |


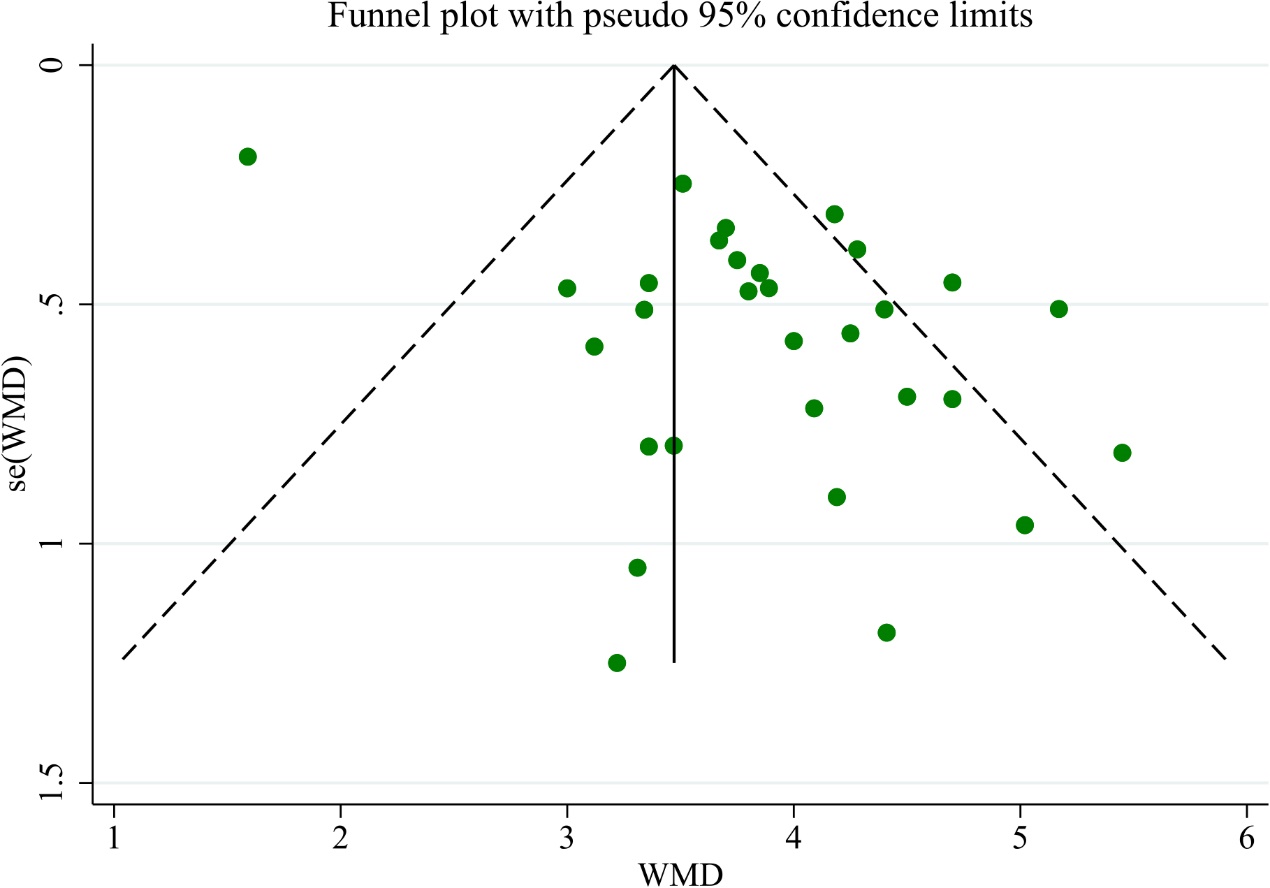


**Supplementary Figure 1: Results of Publication Bias Detection.**
